# Reducing Operational Expenses of LoRaWAN-Based Internet of Remote Things Applications

**DOI:** 10.3390/s22207778

**Published:** 2022-10-13

**Authors:** Mariano Finochietto, Rodrigo Santos, Sergio F. Ochoa, Roc Meseguer

**Affiliations:** 1 Grupo de Investigación en Desarrollos Informáticos, Department of Information Technology, Universidad Nacional de Mar del Plata, Mar del Plata B7600, Argentina; 2 Department of Electrical Engineering and Computers, Instituto de Ciencias e Ingeniería de Computación (CONICET-UNS), Universidad Nacional del Sur, Bahía Blanca B8000, Argentina; 3 Department of Computer Science, Universidad de Chile, Santiago 8370456, Chile; 4 Department of Computer Architecture, Universitat Polytecnica de Catalunya, 08034 Barcelona, Spain

**Keywords:** LoRaWAN extension, Internet of remote things, IoRT-based monitoring systems, remote sensing, reduction of operational expenses

## Abstract

LoRaWAN has become the most widely used low-power wide-area network technology to implement monitoring solutions based on the Internet of remote things (IoRT) paradigm. Typically, these solutions interconnect remote sensing areas and data processing infrastructure located in urban centers. The operation expenses of these solutions depend mainly on the traffic sent through the network backhaul, i.e., the link that connects the remote sensing area and the urban area where the data are usually processed and stored. This service is provided by telecommunication companies and represents the main operation cost of IoRT solutions. These expenses usually limit the affordability of IoRT-based systems in developing countries, and also in scenarios where the operational cost is an issue to address. This paper presents an extension to the LoRaWAN protocol, named Node-Aware-LoRaWAN (NA-LoRaWAN), that reduces the traffic in the backhaul, thus decreasing the operational expenses of IoRT-based systems. In order to evaluate the performance of NA-LoRaWAN, it was compared to a regular LoRaWAN implementation. Depending on the network scenario, the proposed extension reduced the traffic through the backhaul in the range of 12–34%. This extension opens several opportunities to use IoRT solutions in application domains with a low operational budget, e.g., precision agriculture, environmental monitoring and natural hazards’ early detection.

## 1. Introduction

The Internet of remote things (IoRT) [1] is a branch of the Internet of things that studies the interconnection of devices that operate in remote areas. Many IoRT applications have been designed to support remote monitoring in rural areas, for instance, in oil or mining operations, where the sensors measure performance variables of the extraction machinery, to help remote experts predict failures, monitor the operations and make on-time decisions. IoRT applications are also used in precision agriculture and farming, for instance, in irrigation control and cattle supervision.

Typically, the monitored areas involve hundreds of sensing devices that send data to urban centers where such data are processed and stored. The sensing and the processing areas are connected through a stable link, known as the backhaul, that is a service usually provided by telecommunication companies.

In many application domains, the traffic on the backhaul represents an important operational expense to the owners of IoRT-based systems; for instance, small farmers, nonprofit organizations, and rural communities that use monitoring and forecasting systems, early warning systems and diagnose applications remotely to support several daily activities.

This expense increases its impact in developing countries, where the communication through the backhaul is usually limited to a maximum traffic per month (data cap). For instance, SWARM, a well-known IoT satellite communications service, limits the usage of its channel to 750 messages per month (up to 192 bytes each) [2]. This may be fine for a single device, but not for an IoRT gateway that receives data from many remote sensors.

Other options, such as Starlink (a satellite Internet provider without data cap), provide a service that is two to eight times more expensive than the ISPs found in urban areas [3,4]. In the middle, there are other options, such as Inmarsat BGAN M2M, which offers different data plans starting at 2 MB per month [5]. Although the price/MB is more expensive than Starlink, the monthly bill could be lower if the network usage is low enough.

Regardless of the traffic expenses and data cap for using the backhaul, in developing countries, the terrestrial telecommunication infrastructure used to implement this segment typically provides unstable communication links to the Internet [6]. Frequently, this backhaul is also implemented using obsolete satellite communications or cellular technologies (e.g., 2G) [7]. In these cases, the network usage is restricted either by its high price, or due to bandwidth or data cap. Therefore, it cannot be assumed that an IoRT-based application that works smoothly in an urban area will keep working in the same way, with the same bandwidth and with the same operational expense when sensing components are located in rural areas.

Recent low-power wide-area networks, such as Sigfox, NB-IoT or LoRaWAN, have appeared as suitable options to support the communication in IoRT systems, particularly when the network has to connect urban and rural areas. These networks use low-power consumption devices, which is a key feature to make feasible the operation of solutions in scenarios where a stable source of energy may not be available. Moreover, they can offer a communication coverage of a wide area using very little infrastructure; e.g., the communication threshold of a gateway can reach 10 km [8].

In some cases, particularly when the distance between the sensors and end-users is not too long, these networks avoid the use of a backhaul, thus reducing the operational expenses of IoRT systems. However, in large rural areas, such as those covering Latin America, using only these networks is not enough to avoid using a backhaul.

In long-term monitoring applications where it is difficult to replace batteries or provide technical support to devices, the introduction of traffic-shaping policies that can prolong battery life is an important issue, especially for the gateways or concentrators that collect the information in the field to upload it to data processing centers. In [9], a study was conducted on how the prediction of topology control messages in human-centric wireless sensor networks can be used to help reduce the energy consumption of the participating devices; in that work, there were machine-to-machine communications.

Considering the described scenario, this paper presents an extension to the LoRaWAN protocol, which does not avoid the use of the backhaul, but reduces the traffic on it. This extension, named *Node-Aware-LoRaWAN* (NA-LoRaWAN), decreases the regular operational expenses of IoRT systems compared to LoRaWAN.

The Section 2 presents some background on LoRaWAN networks. Section 3 discusses the related works. Section 4 describes the NA-LoRaWAN proposal, including its structure and behavior. Section 5 shows how to implement NA-LoRaWAN. Section 6 describes the simulations designed to evaluate the proposal and the obtained results. These results are then used to compare the traffic in the backhaul using both LoRaWAN and NA-LoRaWAN. Section 7 discusses the evaluation results, and Section 8 presents the conclusions and future work.

## 2. LoRaWAN Networks

**Figure 1:**
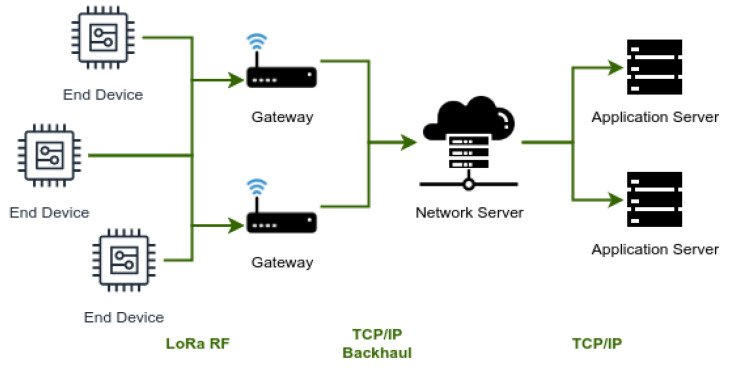
Diagram of a typical LoRaWAN network scenario.

LoRaWAN is an open communication protocol designed to connect IoT devices to a backend that is usually located in the cloud or a remote server. This protocol is composed of a physical layer that uses a radio modulation called LoRa, based on chirp spread spectrum, and a MAC layer that defines an open-source networking protocol. The structure of a LoRaWAN network involves four types of nodes (Figure 1):

1. *End devices:* These are sensors or actuators that receive and transmit data from/to the gateways using LoRa modulation. They are usually located in remote areas.
2. *Gateways:* They are edge nodes that forward packets between end devices and the network server. They act as a message bridge between the LoRa radio and the backhaul protocol.
3. *Network Server:* This node is in charge of registering gateways and end devices and managing the whole network. Typically, a LoRaWAN network has only one of these components, that is usually located in urban areas.
4. *Application Servers:* These components store and process the information received from the sensors and provide services to end-user applications, software agents and IoT devices, i.e., these application servers represent the backend of the IoRT systems for service consumers or client applications.

The activation of end devices in the network is done using activation by personalization (ABP) or over-the-air activation (OTAA). In ABP, the device to be activated should be already configured with a fixed address and session keys (needed to establish communication) before installation. In OTAA, the configuration parameters are dynamically assigned in a process called join procedure, which involves the participation of the network server.

Once activated, the devices use the network structure shown in Figure 1 to exchange data or commands with other devices. Particularly, the gateways transmit to the network server every data packet received from the end devices.

By protocol design, there is no standard way for a gateway in LoRaWAN to associate a packet with a unique end device (i.e., data source or end point). This job is done by the network server, through a process that matches each message’s cryptographic signature to a device in a backend database. Since the gateway does not know the cryptographic key used (network session key), and it does not have access to the devices database, these nodes cannot match the message with a device; therefore, they cannot apply a packet-filtering policy based on the source node id.

Alternatively, the gateway could use the *DevEUI* to identify end devices. This is a unique identifier assigned by the chip manufacturer, similar to the MAC address. However, as it is only sent during the join process in OTAA, it is not accessible in future packets transmissions, neither in OTAA nor in ABP, and hence, the gateway does not know the DevEUI of each incoming data packet.

Finally, the gateways could use the *DevAddr* to identify end devices, and thus implement policies to filter packets before sending them to the network server. The DevAddr is an address either hardcoded in the device (when using ABP) or assigned by the network server (when using OTAA). Therefore, there may be various devices with the same DevAddr in the network, which makes this address useless to identify nodes. However, if the DevAddr assignment process is changed to guarantee that each node id is unique, then, in some scenarios, the use of this attribute would be sufficient. For instance, in networks where the nodes have none or little mobility (as the ones targeted by this work), i.e., the nodes that are always within the communication threshold of the same gateway. In these cases, the DevAddr is present on every packet that passes through the gateway; therefore, it is possible to identify the end devices.

On the other hand, making gateways filter incoming packets renders roaming unusable, since LoRaWAN roaming does not support private networks. Nevertheless, for the use cases targeted by this work, the roaming is not a relevant aspect in the network design.

## 3. Related Work

The challenges of adapting the LoRaWAN protocol to work in networks with limited backhaul has been the focus of several research studies. For instance, in [10], the authors identified three open issues: the *network latency* that may be greater than ACK timeouts, the *packet duplication* that may load the backhaul with multiple copies of the same message and the *data overhead* that serialization protocols introduce at the application layer. This latter challenge was also analyzed in [11,12,13], where the authors proposed to address it using a compression technique based on protocol buffers. This strategy of reducing the size of the packets sent through the backhaul is complimentary to others approaches, such as avoiding sending duplicated packets.

In [14], the researchers introduced an architecture for LoRa-SDN (software-defined networks), which set the basis for processing packets at the gateway level and made network administration easier and more scalable. In that model, an SDN software switch was installed in every LoRa gateway to filter packets. In addition, an SDN controller was installed together with the network server to synchronize the SDN switches. Even if the general model could work with LoRa, its design would not be compatible with LoRaWAN, since the architecture assumed that the network could identify end nodes at the gateway level, which cannot be done using the regular protocol. In [15], the authors proposed LoRaMoto, which extended the LoRaWAN architecture and implemented a packet-forwarding mechanism between the end nodes of the system to provide alternative-path communications.

Fog computing in LoRaWAN has also been the subject of research. In [16,17,18], the authors proposed two groups of architectures which, though valid for several scenarios, were not suitable to support IoRT-based solutions. First of all, the LoRaWAN network and application security keys had to be downloaded and processed by the gateways, which compromised the system security. Second, some components of the application stack had to be installed in the gateways deployed in the field, which represented an important operational cost. Moreover, having a thick processing layer in the gateways would require more computational resources and a higher energy consumption in the field.

Similar to the previous architecture, other researchers have proposed different approaches for connecting LoRaWAN networks through satellite [19,20], for instance, to support remote monitoring of wind farms and massive machine-type communication. Particularly, in [21], the authors included preliminary ideas to implement *direct* and *indirect* links, where the first one involved putting a LoRaWAN gateway in the satellite, and the indirect links were used to support interactions between a LoRaWAN gateway and the satellite backbone.

Other research works, such as those reported in [22,23], aimed to optimize traffic and devices’ energy consumption by assigning a QoS to the nodes depending on the requirements of the applications. These proposals were mainly focused on adjusting the setup parameters of the radio transceiver in end devices and gateways, which is a research aspect that is out of this article’s scope.

In order to reduce the traffic through the backhaul in IoRT systems, the next section describes the proposed extension to LoRaWAN (i.e., NA-LoRaWAN). This extension is based on a previous work, where the authors proposed an agent that processed packets in an intermediate node [24], i.e., after the packet had been sent by the source node, and before sending it at the destination. This agent was able to filter, aggregate or transform data dynamically.

## 4. Structure and Behavior of NA-LoRaWAN

This proposal extends the structure and behavior of the regular LoRaWAN gateway and network server. Moreover, it allows the coexistence of regular and extended LoRaWAN components in a same network. Therefore, the main difference between a regular LoRaWAN network and others that use the extended components, is that the latter have the capability to manage the traffic sent through the backhaul, i.e., the traffic between the gateways and the network server. The larger the number of extended components used in network, the more the capability of the system to reduce the traffic sent through the backhaul.

**Figure 2:**
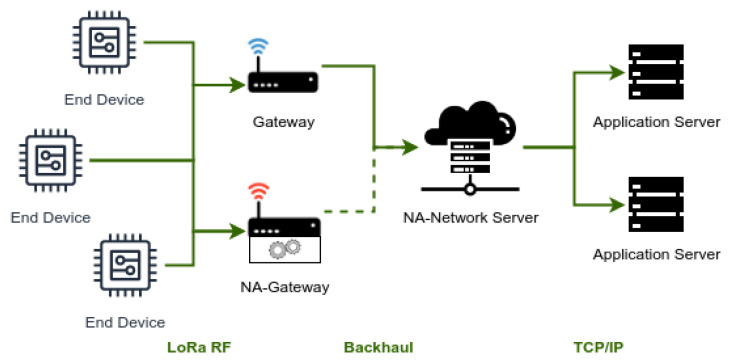
Architecture of a hybrid LoRaWAN scenario. Blue signal indicates traditional LoRaWAN gateway and red signal indicates a NA-LoRaWAN gateway.

Figure 2 shows a network architecture, similar to the previous ones, but now including an NA-gateway and NA-network server, i.e., the extended versions of the regular ones.

In order to reduce the traffic through the backhaul, every NA-gateway uses a packet filtering policy that is indicated by the NA-network server according to the network conditions. Considering the filtering policy in use, the NA-gateway analyzes the incoming packets and determines whether each one must be sent to the NA-network server. A packet is discarded only when the NA-network server has the capability of receiving it through another source, and thus the network reduces the traffic through the backhaul.

The contrast between the behavior of regular and extended gateways is illustrated in Figure 2, where the gateway at the top (regular one) forwards every incoming packet, while the gateway at the bottom (extended one) processes the packets and only forwards some of them.

**Figure 3:**
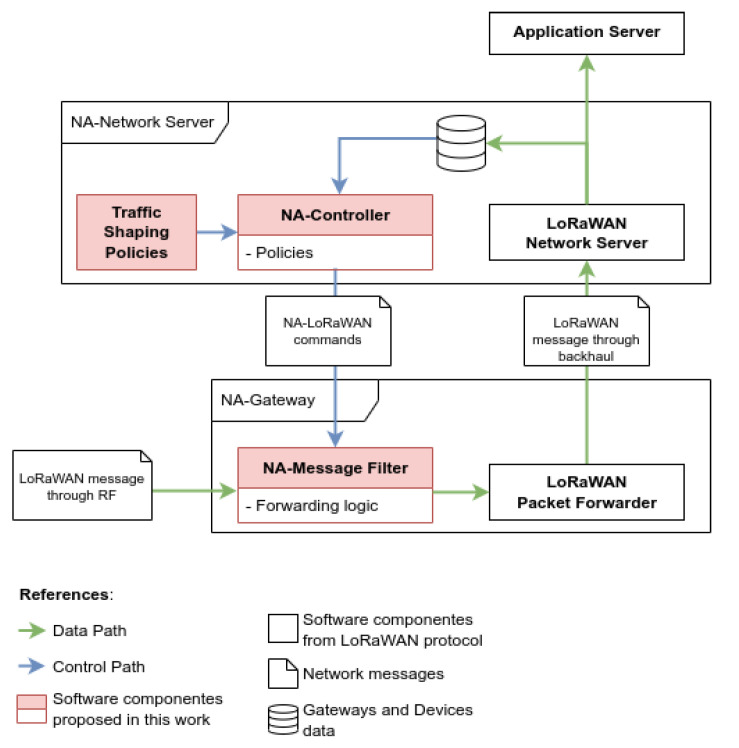
Communication process between the NA-gateway and NA-network server.

Figure 3 illustrates NA-LoRaWAN in more detail and explains the dynamics of the communication process between the NA-gateway and NA-network server. Particularly, the green lines indicate the data transmission process, and the blue ones show the flow of control. Next, we introduce the NA-gateways and NA-network server.

### 4.1. The NA-Gateway

Regular gateways are packet forwarders; however, the NA-gateways include the NA-message filter component that determines if a certain packet should be forwarded or discarded, according to the filtering policy used by that node. For a gateway to be *node-aware*, it needs first to uniquely identify which node has sent an incoming packet, and then apply filtering rules to decide what must be done with this packet—either forward it or discard it. Having the ability to filter packets by node id allows the NA-gateway not only to apply filters based on the sending node, but also to classify devices into clusters, and process them based on that classification (e.g., to create priority levels and treat each level in a different way).

Periodically, the NA-message filter receives updates from the NA-controller with the information that it needs to operate. Whenever a command is received, it runs the traffic-shaping policy indicated by the NA-network server. Given these policies are specified as a set of ordered rules (e.g., implemented in a script), they can be delivered using regular messages to the NA-gateways when required.

### 4.2. The NA-Network Server

As shown in Figure 3, the NA-network server includes a network server and the messages repository (that are part of regular LoRaWAN), but it also adds the NA-controller and a set of traffic-shaping policies to filter packets. Provided that there is only data coupling between the NA-controller and the network server, both software components can be considered almost independent from each other. However, for robustness and performance reasons, it is recommended to run both components on the same computer.

The NA-controller is allowed to read data from the server repository. Using such data, the controller diagnoses the traffic that is arriving at the network server and determines if it needs to adjust the policies used by some NA-gateways. When a change is required, the NA-controller informs each particular NA-gateway which new filtering policy it has to apply (shown in blue lines in Figure 3).

These commands are sent to the gateways only in particular instances, e.g., whenever a new node needs to be activated (once at the beginning, normally), when a node is moved or removed, and when it must change its filtering policy. Consequently, if the network infrastructure does not change frequently, as expected in IoT solutions, these commands will have a negligible impact on the traffic backhaul. For this reason, this aspect is not formally addressed in this work, but it is discussed later on.

## 5. Implementing NA-LoRaWAN

In NA-LoRaWAN, the end devices do not need to be extended to work indistinctly with NA-gateways and regular ones. As mentioned before, an NA-controller should be installed together with the network server, and also an NA-message filter should be part of the NA-gateways. All communication between network server and an NA-gateway is done between the NA-controller and NA-message filter.

**Figure 4:**
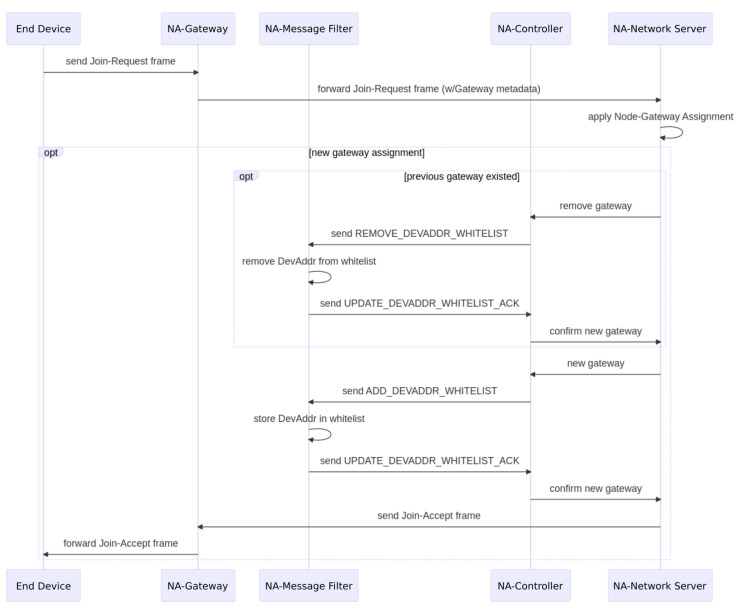
Gateway whitelist updating sequence.

Typically, the end devices are incorporated into the network by linking them to the gateway with a better connection. For this, the NwkAddr field is used for the DevAddr. The sequence diagram shown in Figure 4 illustrates this process.

Particularly, the network server exchanges messages with the NA-message filter in the NA-gateway to generate the whitelist of end devices that are forwarded through the backhaul. Each NA-gateway that listens to an end device will not forward its incoming messages until its NA-message filter acts.

Provided that all NA-gateways can operate as regular gateways, the end devices should be compatible with both gateway types. Moreover, an NA-gateway should be able to become NA or a regular gateway on the fly, once it receives an *activation* or *deactivation* command from the NA-controller. This allows the network server to find the right balance between packet redundancy and traffic optimization. The structure of the command packets is shown in Table 1.

Once an NA-gateway is deactivated, it locally keeps all data already related to this mode, since the gateway can change the mode again in the future. Moreover, in both modes, the gateway should process and respond to every command from the NA-controller. To erase the gateway data, a RESET command must be sent by the controller.

In order to mitigate the packet loss caused by disconnected gateways, when one of them goes down, its neighboring gateways keep transmitting all messages providing redundancy. Gateways inform their status to the network server by sending a heartbeat or keep-alive message, which is a common technique already implemented in popular LoRaWAN stacks such as ChirpStack.

When an NA-controller detects that a gateway is down, it deactivates all NA-gateways, transforming them into packet forwarders. Thus, the packets assigned to the disconnected gateway have the chance to be forwarded by other gateways. In this situation, packets reception by the network server is prioritized over backhaul traffic optimization, and the fault tolerance strategy becomes the one standard in LoRaWAN.

Then, if the disconnected gateway becomes operational, the NA-controller activates back the NA mode into the corresponding gateways. When the network infrastructure changes because some gateways are removed or added, the network manager can do a manual or automatic update. In any case, a reconfiguration of the network is done by updating the DevAddr of end devices, and eventually reassigning them to the gateways.

### 5.1. Filtering the Backhaul Traffic

This section indicates how to implement four traffic-shaping policies or profiles on NA-LoRaWAN and manage the downlink messages sent through the backhaul. These messages include the node id, node priority, number of packets, and budget. Each profile has a particular purpose while reducing the traffic on the backhaul. The network administrator handles the knobs of the profiles by considering a weighted sum of the policies, where each one is multiplied by a particular coefficient. In this way, an operational index (OI) can be defined to indicate the way in which the backhaul behaves:

(1) $$\begin{array}{cc} OI & =\alpha P_{1}+\beta P_{2}+\gamma P_{3}+\delta P_{4} \end{array}$$

where OI is the operational index, $P_{i}$ represents each particular policy, and α,β,γ, and δ are the knobs of the system. OI is always equal to 1, and it is obtained by modifying the coefficients of each policy, i.e., setting the knobs. An appropriate setup of these coefficients should be made based on the network administrator’s experience. In this way, when the number of packets has reached its maximum, γ=1, forcing all the other coefficients to zero. Therefore, no more packets are sent.

#### 5.1.1. Filtering by Node Id

Each NA-message filter at the gateway could keep a local whitelist of DevAddr (similar to a flow table in SDN) that is processed to determine if a packet should be forwarded to the network server. This list will be updated by the NA-controller and corresponding NA-gateway when the former informs that a new ABP device has been configured by the network administrator, or each time a new device concludes a successful join process in OTAA.

If two gateways notify the NA-controller of a join process (OTAA) of the same node, then the NA-controller will choose the one having the lower RSSI. In the case of ABP, it is up to the network administrator to decide which gateway will transmit the packets of each end device.

The NA-message filter broadcasts its status periodically (e.g., once per day), and the NA-controller keeps a log of the status of every gateway. If a gateway fails to relay its status and it is not sending packets anymore, the NA-controller could migrate or distribute the node’s whitelist to nearby NA-gateways. By maintaining a whitelist of end devices without repeating DevAddr between them, this allows gateways to transmit each message to the network server only once, thus reducing energy and operational costs.

**Figure 5:**
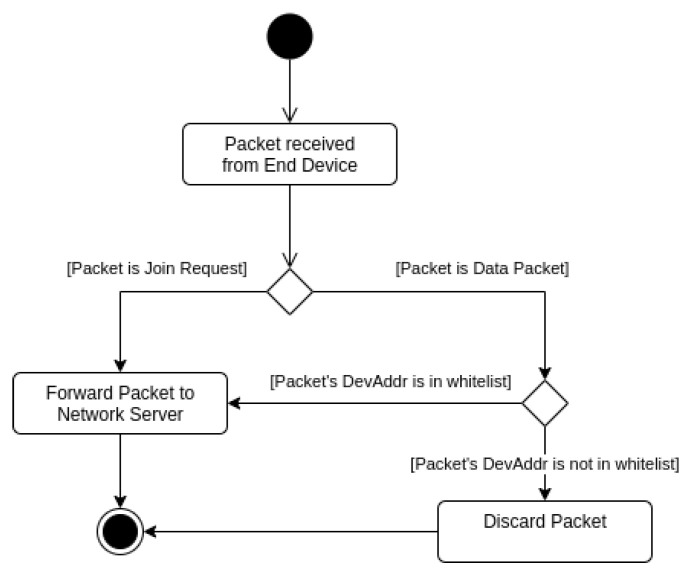
Packet filtering at the gateway level.

When an NA-LoRaWAN gateway receives an incoming packet, it should first check whether it is a join request or a data packet (Figure 5). For the first case, the request should be forwarded immediately. For the second case, the gateway should first check if the DevAddr in the packet is already in its whitelist. If it does, then the packet is forwarded; in other case, it is discarded.

#### 5.1.2. Filtering by Node Priority

The NA-controller can assign a priority level to each end device (e.g., levels 1 to 5, 1 being the most important node, and 5 the least important one). Then, when the controller needs to save network bandwidth, it can instruct the gateways to only forward packets from nodes with a certain priority level. By default, every node has the same priority, and every gateway forwards messages from any node. However, these two properties can be changed by the network administrator. Every time a priority level to forward packets is changed, the network server informs it to each NA-gateway.

This priority should be assigned by the system manager to each end device based on the use case requirements. A priority field, sent together with the DevAddr in the ADD_DEVADDR_WHITELIST packet payload, indicates the priority level of the end device. Moreover, a SET_PRIORITY packet should also be sent during network setup to inform the priority level to filter. For example, if the payload of the SET_PRIORITY packet indicates level 1, then all packets coming from nodes with level 2 or above will be discarded.

This approach is not strictly a real-time approximation [25] but may be used to schedule traffic with time restrictions with a higher priority in an expedite way.

#### 5.1.3. Filtering by Maximum Number of Packets

In this case, the gateway receives from the network server the maximum number of packets (data cap) allowed to be forwarded in a certain time period. This information is transmitted in the payload of the SET_MAX_PACKETS packet.

The gateway should locally filter packets of an end device if the node has already sent all allowed packets during one period (specified in minutes, hours, or days). If there is no period information, the gateway should forward all packets.

A scenario in which this policy could be used is when the network provider offers a limited number of packets to transmit through the backhaul. It can also be used when the application server does not require to read every incoming packet, but only some of them periodically. For instance, an application could require getting one temperature sample from a device every one hour, but the sensors in the field are transmitting such information every 30 min. In that case, half of the packets are not consumed by the application, so they can be filtered before being forwarded.

When LoRaWAN applications are registered, the application server could optionally indicate the period and number of packets the end devices are allowed to transmit. If this information is not filled in, this server assumes the device can send data at any time.

This information is then aggregated for all devices, and downloaded to all the NA-message filters. Therefore, if a node is sending messages at a frequency higher than the one that is allowed, the gateway will filter those extra packets locally.

#### 5.1.4. Filtering by Budget

This filtering policy uses a logic similar to the previous one but using a budget that limits the number of bytes to be forwarded. In this sense, each gateway receives a SET_BUDGET packet from the network server, containing in its payload the maximum budget allowed to be forwarded. Every time a gateway receives a packet from an end device, it applies the logic described in Section 4.1 and decides to forward or discard the packet.

The size of the budget is a design decision that can be different depending on whether it corresponds to a gateway or an end device. Moreover, the period associated to the budget may also vary from minutes to days, depending on the use cases. If there is no critical information being sent by end devices, then the NA-controller could limit the number of bytes sent through the backhaul, by reducing the budget per node or per gateway.

Each time a gateway receives a packet, it first checks if the available budget is bigger than the packet size. If so, then the gateway subtracts such packet size from the corresponding budget. When the available budget is smaller than the packet size, the packet is not forwarded, but rejected.

#### 5.1.5. Filtering Downlink Messages

In order to reduce the backhaul traffic, managing efficiently the downlink messages is also relevant. Typically, these messages come from software systems that use the application servers as backend. In these cases, it is enough to add logic in the application (in frontend or backend) to optimize the use of downlink messages, for instance, send these messages when extremely necessary. It does not affect the protocol behavior; therefore, the NA-gateways do not filter the incoming downlink messages from the NA-network server.

## 6. NA-LoRaWAN Evaluation

In order to evaluate the performance of NA-LoRaWAN and determine its potential benefits, the NA-gateways, the NA-network server and the described traffic-shaping policies were simulated. This section describes how these simulations were carried out, as well as the obtained results.

### 6.1. Simulations Setup and Parameters

The simulations were implemented using the NS3 LoRaWAN module described in [26,27,28]. This module was modified and extended to add an extra logic layer, which was required to run the gateways’ filters. The resulting code is available in a public repository [29]. Next, we describe the simulation scenarios and parameters used in the evaluation process.

#### 6.1.1. LoRaWAN Configuration

The LoRaWAN parameters were meant for Europe, where the NS3 module was developed. Particularly, the transmission power was 14 dBm (maximum for Europe) in the EU863-870 channels. The bandwidth and data rate were the default ones (125 kHz and DR0 to DR5). The spreading factor (SF) was not set because the simulation calculated the optimal one for each device, by making sure it reached the minimum quantity of gateways. For this simulation, this represented a worst-case scenario, since the evaluation goal was to determine how efficient this proposal was in terms of traffic optimization.

#### 6.1.2. Simulation Scenarios

Clearly, it is not feasible to get general results that apply to any use case, since there are several factors affecting the network behavior. These factors go from gateway and end devices’ locations to qualitative data analysis (e.g., assigning priority to packets in every traffic-shaping policy). Therefore, the simulations were run over 100 scenarios created with different but arbitrary settings. The scenarios were divided into five groups of 20 instances each, depending on the number of gateways: 1, 2, 4, 8 and 16 gateways.

All scenarios had a random number of end devices from 10 to 2000. These devices were randomly located following the pseudorandom number generator function *Math.random()*, provided by Node.js v14 runtime.

Given this extension was conceived to support small private LoRaWAN networks, it was decided to work with less than 2000 end devices and 16 gateways. Priority levels from one to five were assigned to end devices in a uniform way, particularly for evaluating the “filter by node priority” traffic-shaping policy.

**Figure 6:**
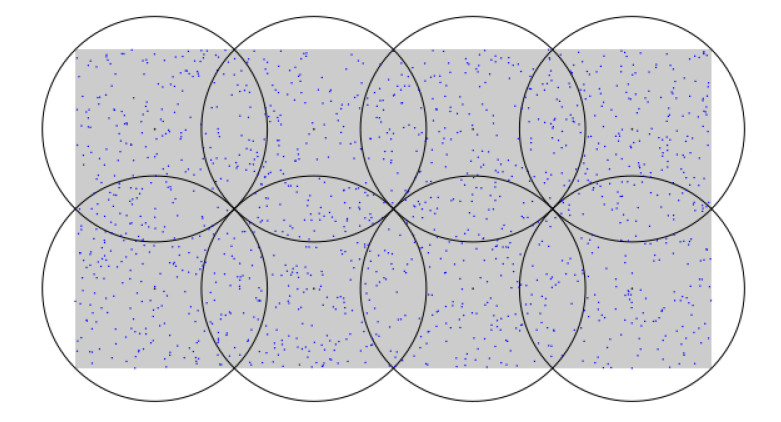
Sample scenario used in the simulations.

The gateways were positioned following a grid pattern as proposed by [30], which was shown to be an efficient algorithm to reduce the number of required antennas. The coverage of each gateway was around 9 km, which was determined by the propagation model proposed in [26]. This simulation scenario did not take into account buildings interference, as the use cases targeted did not involve sensing in urban areas. Figure 6 shows an example scenario with eight gateways, where the black circumferences represent the estimated area of coverage for each gateway. The blue dots represent the dispersed end devices. The gray rectangle in the background delimits the area where the end devices can be located.

The figure shows that some nodes were in the reach of more than one gateway. However, whether the gateways were able to receive packets from a node or not depended mainly on the SF, which was adjusted automatically by each end device to reach at least one gateway. Therefore, it was more suitable to run a simulation to count duplicated packets, rather than just counting the number of nodes inside a certain gateway coverage area. The simulations also considered the NA-network server located in the cloud, and that each NA-gateway had a link to the backhaul network.

#### 6.1.3. Considerations on Control Packets

Each simulation lasted one hour, and hence, initialization packets of both the LoRaWAN specifications (such as join requests) and the ones introduced by this work (such as the updates to the gateway whitelist), were not taken into account since they had a negligible impact on the traffic sent through the backhaul. These initialization packets were only sent at the beginning of the process, and under stable working conditions their impact became imperceptible over time.

Moreover, in order to simplify the simulation, it was determined that all packets were uplink and sent every 20 min in unconfirmed mode, with the random phase provided by the NS3 LoRaWAN module. As a final remark, all packets had a size of 19 bytes and since this module did not encrypt packets, they were significantly smaller than real ones.

#### 6.1.4. Recorded Metrics

In each simulation, the following three metrics were recorded:

- *Rx ratio*: The total packets transmitted by the gateways over the total packets received by them.
- *RxUnique ratio*: This ratio is similar to the previous one, but considers only unique packets transmitted and received by the gateways.
- *RxDuplicated ratio*: This is similar to the previous ones, but it computes only the duplicated packets.

The *Rx* prefix in the metric name represents the point of view of the network server. However, since it was assumed that there was no packet loss between the gateways and the network server, their values were exactly the same as if the transmission from the gateways were counted. In the description of the results, we use indistinctly *Tx* from gateways or *Rx* from the network server.

Duplicated and unique packets were considered across all gateways. Therefore, if two different gateways received the same packet, one of them was counted as unique and the other one as duplicated. We measured unique and duplicated packets to understand how many unique data the protocol was losing, as a consequence of the backhaul traffic reduction.

#### 6.1.5. Simulation Baseline

In the first round, we simulated both gateways using the four traffic-shaping policies and then forwarding any packet to the network server. In total, 100 scenarios were generated resulting in 500 simulations with more than 1,500,000 packets transmitted. The simulation settings were the following:

- *Budget of bytes to forward*: We set that 19 bytes per node could be sent every hour. This was equal to the size of one packet. If more bytes were needed to be transmitted, these packets were discarded.
- *Maximum number of packets to forward*: This number was set as one packet per hour; if extra packets arrived, then they were discarded.
- *Priority level*: Level one (level two to five were filtered by the NA-gateways).

### 6.2. Evaluation Results

**Figure 7:**
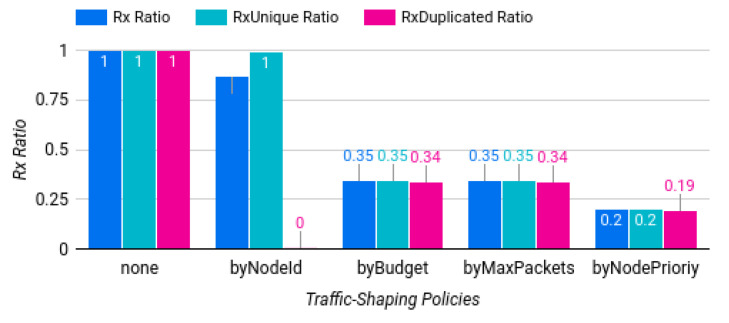
Summary of the baseline simulation results.

Figure 7 shows the summary of the evaluation results of the baseline scenario, which were quite similar to the expected values. The first three bars involve just packet forwarders (i.e., deactivated NA-gateways). The results also show that all packets were transmitted, both unique and duplicated. When using the “filter by node id” policy, only unique packets were transmitted, which in overall represented 87.32% of the total number of packets received by the gateways. Therefore, we could assume that there was an average saving of 12.68% of the bandwidth in the backhaul link.

When using the “filter by budget” and “filter by number of packets” policies, the savings were around 34.57% in the three metrics. This meant the gateways filtered two out of three packets received during the one-hour simulation.

Finally, the “filter by node priority” policy showed that 19.88% of the packets were transmitted, which directly correlated with allowing only packets from priority level one to be forwarded, filtering levels two to five, out of a uniform priority distribution. The next subsections show the performance of the network when changing some particular parameters.

#### 6.2.1. Varying the Number of Gateways

**Figure 8:**
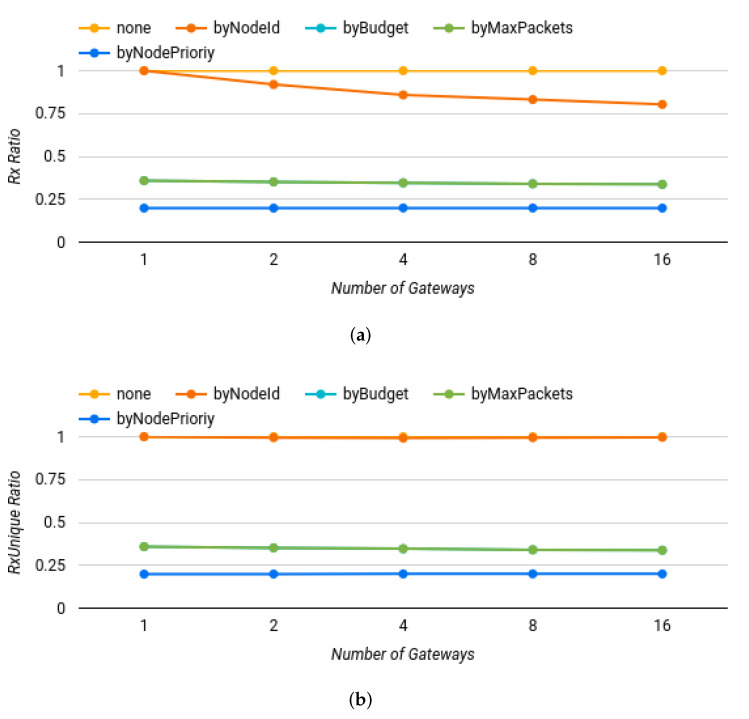

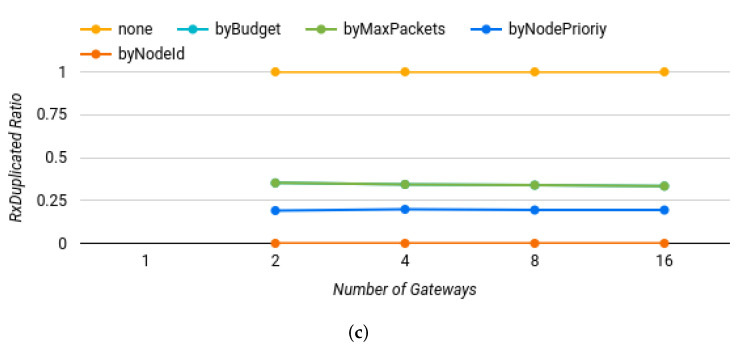
Summary of the baseline simulation results, considering number of gateways and metrics. (**a**) Rx Ratio, (**b**) RxUnique Ratio, and (**c**) Rx Duplicated Ratio.

The grid pattern used for locating gateways considered a strict correlation between these nodes and the size of the scenario. In order to evaluate if the scenario size affected these results, the output was divided into three charts, one for each metric, and grouped by the number of gateways in the *x*-axis. Figure 8 shows the results of the first group of simulations, discriminated by number of gateways and metrics: (a) Rx Ratio, (b) RxUnique Ratio, and (c) RxDuplicated Ratio. The values of series *byBudget* and *byMaxPackets* are similar in the next three charts, therefore they cannot be visually differentiated (they are overlapped).

All policies had similar results as the ones shown previously, except for the “filter by node id” policy (Figure 8a), where the percentage of packets transmitted decreased as the scenario got larger; it went from 100% when there was only 1 gateway, to 80.31% when there were 16 gateways. In (Figure 8b), the series *none* and *byNodeId* are both with value equal to one.

Considering these simulations in context, it can be concluded that the “filter by node i” policy improved bandwidth savings (as more gateways were used), without affecting data loss. The other policies were consistent and scaled with more gateways.

#### 6.2.2. Varying the Priorities

As explained previously, end devices had a five-point priority (one to five) that was assigned in a uniform way. In a new set of tests, the priority level was incremented in every execution, starting from zero (no packets are forwarded) until five (every packet is forwarded). For simplicity, these tests were run only in the 20 scenarios generated involving four gateways. All other settings, apart from the priority, remained the same as the previous ones. A total of 100 simulations were run with more than one million packets.

**Figure 9:**
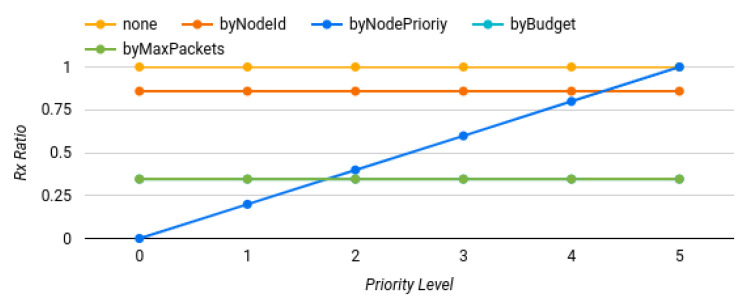
Results when varying the priority level.

Only *Rx Ratio* is shown in Figure 9, since results for *RxUnique* and *RxDuplicated* were similar. It shows, as expected, that only the policy involving priority threw different results in every test. As in the previous cases, the *byBudget* and the *byMaxPackets* lines in the Figure 9 overlapped.

The first execution had 0% of packets transmitted, the second one had 19.94%, the third one had 39.93%, the fourth had 59.83%, the fifth had 79.91%, and in the last one 100% of the packets were transmitted. The slope of the line was incremented 1/5 approximately, which correlated to the five priorities assigned. From these results, it can be assumed that for the priority policy, the trade-off of unique and duplicated packets was directly proportional to the priorities configuration.

#### 6.2.3. Varying the Maximum Number of Packets

In order to determine the impact of the proposed extension, another set of tests were run. They involved changing the maximum number of packets per end device to be forwarded by a gateway in a one-hour time period. One packet per hour was used in the first test, two packets in the second one, and finally, three packets. Again, only scenarios with four gateways were run and the settings were similar to those of the previous tests.

**Figure 10:**
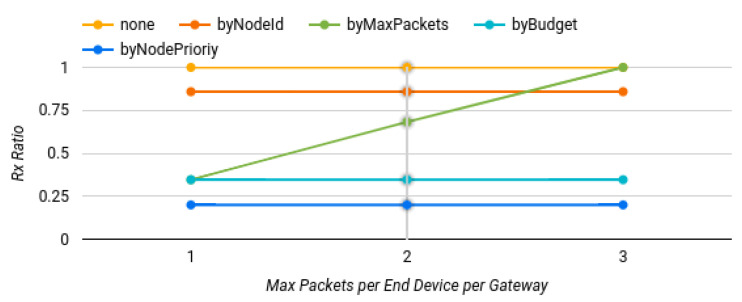
Results of varying the maximum number of allowed packets.

The results shown in Figure 10 indicate that only the “filter by maximum number of packets” policy had different outputs in every run of the tests. As expected, in the first test one-third of the packets were transmitted (34.62%), two-thirds in the second tests (68.20%) and all of them in the last test.

#### 6.2.4. Varying the Budget

Using an approach similar to the previous tests, we analyzed the budget allowed to be transmitted per node per gateway in a one-hour time period. This meant that whenever a gateway received a packet from an end device, it checked whether if it had any budget to forward that packet.

For the first test, as before, the same 19 bytes were used. Then, it was increased to 38 bytes, and finally to 57 bytes. As all packets had a default size of 19 bytes, in the first budget, only one packet could be sent, while in the second and third cases, two and three packets could be transmitted, respectively. Apart from the budget, the other settings were the same as those of the previous simulations.

**Figure 11:**
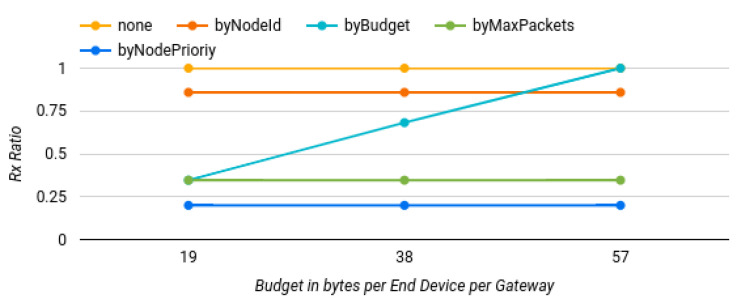
Results of varying the allowed budget in the different traffic-shaping policies.

The results in Figure 11 show that the only policy affected by the budget change was the “filter by budget”. As it was expected, in the first case almost one-third of the packets were transmitted (34.62%); in the second case, almost two-thirds (68.20%); and finally, in the last case all of them were sent.

## 7. Discussion

The simulation results indicated that there was a reduction of the traffic in the backhaul when using NA-LoRaWAN. Such a reduction could be substantial depending on the use case scenario.

In the “filter by node id” traffic-shaping policy, 100% of the duplicated packets were discarded at the gateway level without losing data. For the other three policies, there was a trade-off between traffic reduction and data loss. The impact of this trade-off depended on each scenario and the policy configuration. Managing this trade-off is in the hands of the network administrator, who has to decide the specific profiles to deal with the traffic through the backhaul, based on the characteristics of each implementation.

It is important to remark that the NA-gateway can handle, at each instant, different operating modes according to the particular requirements of the network system. For instance, if the network provider limits the quantity of messages allowed to be sent per month, and the network is near that limit, the administrator may decide to apply a priority policy, by restricting transmission to the nodes with a higher priority. In this case, the administrator is willing to sacrifice data in favor of traffic reduction, thus keeping the IoRT systems operational.

If instead, all the nodes share the same priority, the administrator may choose to limit the number of packets by applying the corresponding policy. A similar situation may occur if the network provider limits the traffic by bandwidth. In that case, the administrator can apply the limit by budget policy (e.g., by hour or day), which would resample the messages.

A cost performance index (CPI) could be tracked in order to determine whether it is needed to dynamically change the configuration of the traffic policies. The CPI is a metric originally used in project management that indicates whether the budget is being executed efficiently. A CPI greater than one states that one is over budget; a CPI less than one indicates being under budget; a CPI equal to one means that the budget is being consumed efficiently. For a defined time period, the CPI can be calculated as shown in (2):

(2) $$\mathrm{CPI}=\frac{Budget}{Throughput}$$

where the budget is the available bandwidth for the backhaul traffic, that is set by the network administrator (in KB or packets), and throughput is the actual traffic transmitted through the backhaul. An example of a CPI use case is when the system automatically changes the “filter by node priority” traffic policy. If the CPI indicates the system is over budget, the system could decrease the priority level allowed to transmit and thus filter more packets. On the contrary, if the CPI indicates the system is under budget, the system could increase the priority level allowed to transmit, therefore, more nodes would be able to send data to the cloud.

On the other hand, the network server and the gateways have to exchange messages periodically in any case. Setting up the configuration for the traffic-shaping policies’ implementations requires a few additional message exchanges with respect to the regular LoRaWAN operation. The extra cost is justified by the reduction of traffic and the benefits from implementing different packet-transmission policies.

The generalization of these results is limited by the number of end devices and gateways deployed in the field, and also by their locations. First, the evaluation was done based on 100 scenarios, which may not consider all possible cases. For instance, if there is no overlap in the gateways’ reach, then the “filter by node id” policy does not bring any benefit compared to using standard LoRaWAN gateways. Second, there may be more aspects to consider in order to adapt the protocol to limited backhaul networks, e.g., when network latency affects the LoRaWAN timeouts. However, it is important to remark that the scenarios considered in the simulations cover all the typical ones.

On the other hand, the architecture proposed in this LoRaWAN extension can use several traffic-shaping policies. Particularly, when more fault-tolerance is preferable, a duplication of packets can be an option. When high-priority messages should have guaranteed access to the network server, the selection of a priority-based policy is preferable.

Concerning the security aspects, this extension does not modify the security mechanisms of LoRaWAN. All security keys are preserved, and encrypted traffic is transmitted over the network.

## 8. Conclusions and Future Work

This paper presented an extension to LoRaWAN, which was designed to reduce the traffic sent by the gateways through the backhaul during the operation of IoRT-based solutions. The traffic in such a network segment usually represents high operational expenses for owners of IoRT systems. In several application domains, this cost jeopardizes the feasibility of using IoRT-based solutions, for instance, to support small farming, perform the early detection of natural hazards, or simply monitor remote living or inanimate objects. Many nonprofit organizations and civilians are responsible for keeping IoRT systems running, which operate under these restrictions.

The presented extension, named NA-LoRaWAN, added software components to regular gateways and the network server of LoRaWAN, and thus it implemented various mechanisms to manage the message traffic through the backhaul. The article also showed how to do it using four traffic-shaping policies on NA-LoRaWAN.

The potential impact of this proposal was evaluated using simulations, and the obtained results indicated that NA-LoRaWAN outperformed standard LoRaWAN in terms of traffic sent through the backhaul network. Moreover, in each simulation, we showed how the traffic could scale, and the relationship between the data to be transmitted (sensed data) and the network usage, considering different configurations. Whether it is convenient or not to use a particular traffic-shaping policies will depend on the analysis done for each application scenario.

According to the evaluation results, the expected traffic reductions were in the range of 12–34%. However, the results also showed that this percentage could increase significantly by defining policies more specific to the IoRT applications and scenarios to be supported. In this sense, more extensive and in-depth studies are required to identify patterns or rules that can be reused to manage this traffic in particular use cases. Moreover, the use of combined traffic-shaping policies should also be explored, since it could help mitigate some of the potential issues raised in this paper.

## Figures and Tables

**Table 1:** Structure of the command packets.

Node-Aware Version (4 bits)	Packet Type (4 bits)	Timestamp (48 bits)	Payload (Variable Length)
	0 (NODE-AWARE ACTIVATION)		
	1 (NODE_AWARE_DEACTIVATION)		Optional.
0 (current version)	2 (RESET)	Milliseconds since epoch	Contains specific data that depend on the packet type.
	4 (REMOVE_DEVADDR_WHITELIST)		
	5 (UPDATE_DEVADDR_WHITELIST_ACK)		

## Data Availability

https://github.com/marianofino/ns3-lorawan-simulation.
